# Informing the Development of Tailored Antenatal Care Services for Pregnant Adolescents: A Systematic Review and Stakeholder Survey

**DOI:** 10.3390/nu18050727

**Published:** 2026-02-24

**Authors:** Karissa Bjornstad, Emily Dawson, Amir Ali Barket Ali Samnani, Marko Kerac, Amanda Murungi, Stephanie V. Wrottesley, Natasha Lelijveld

**Affiliations:** 1 Department of Population Health, London School of Hygiene & Tropical Medicine, London WC1E 7HT, UKmarko.kerac@lshtm.ac.uk (M.K.); 2 Emergency Nutrition Network, Kidlington OX5 2DL, UKam_samnani@hotmail.com (A.A.B.A.S.);; 3 Mulago National Referral Hospital, Kampala P.O. Box 7051, Uganda

**Keywords:** antenatal care, infant, adolescent, maternal, pregnancy

## Abstract

**Background**: Pregnant adolescents are at higher risk of adverse birth outcomes. Tailoring antenatal care (ANC) to adolescents’ unique needs may be a way to reduce adverse maternal and child outcomes within this population. This systematic review aimed to evaluate ANC services for pregnant adolescents and their impact on maternal and infant outcomes. **Methods**: Two reviewers independently searched five electronic databases (September 2024) to evaluate existing ANC services that are tailored to adolescents and the impact they have on maternal and infant outcomes. Studies were assessed for quality using the NICE quality appraisal tool and a narrative synthesis was carried out to present the findings. In addition, a survey was disseminated through the Global Adolescent Nutrition Network (GANN) to gain further insights into stakeholder views and experiences of tailored ANC for adolescents. **Results**: 11,236 articles were reviewed, with 14 studies included for analysis. Interventions as part of ANC for pregnant adolescents included micronutrient supplementation, supplementary feeding, community-based delivery, group-delivery, tailored nutrition education, and additional support and counselling. Outcomes such as birthweight, preterm birth, and gestational age were reported, with most studies (11/14) demonstrating positive effects. Of 103 survey responses, 100% agreed that ANC for pregnant adolescents need to be delivered in a youth-friendly manner, and 57% indicated that providing both youth-friendly delivery and additional support are crucial. Inclusive and supportive care, tailored educational support, tailored nutrition care, and mental health support were most commonly mentioned as key components for tailored ANC. **Conclusions**: The systematic review and survey data concur in identifying key elements of adolescent-tailored ANC. Some of these have already been shown to be effective; however, due to the high heterogeneity of the study designs, a stronger evidence-base is needed. Specific elements of future ANC packages for pregnant adolescents might include group ANC delivery, community-based services, increased confidentiality measures, mental health support and counselling, health education, and nutrition care tailored to adolescents’ physiological and emotional needs.

## 1. Introduction

Pregnant adolescents (10–19 years) are often overlooked in global health initiatives, leading to inadequate care and support and increasing the risk of adverse outcomes [1,2]. Whilst progress has been made in lowering fertility rates worldwide [3], it is estimated that 21 million adolescents aged 15–19 years old living in low- and middle-income countries become pregnant each year, of which 12 million give birth [4]. Adolescent pregnancy increases the risk of birth complications, including stillbirth, preterm birth, and low birth weight (LBW), as well as higher maternal stigma, economic impacts, and morbidity mortality rates compared to adult pregnancies (20–40 years) [5,6]. Infants born preterm or at LBWs often face difficulties developing cognitive and motor skills as they grow older [7]. In addition, children born to adolescent mothers have an increased risk of being underweight up to the age of five [8]. Adolescents are more vulnerable to these birth complications due to increased biological, social, and economic risks [9,10].

The 2016 Antenatal Care (ANC) model developed by the World Health Organization (WHO) recommends eight ANC visits during pregnancy beginning at 12 weeks and continuing up to 40 weeks to maintain a healthy pregnancy for mother and baby, facilitate an effective transition to positive labour and birth, and achieve positive motherhood [2]. During ANC visits, essential services are provided, such as health education, maternal and foetal assessments, nutritional interventions, and disease prevention and management for mothers and infants.

Adolescents often face cultural and financial barriers to accessing ANC, resulting in missed opportunities to access these essential services [1]. Commonly reported barriers include cost of transport and distance to health facilities [11], as well as shame, blame, and stigma surrounding pregnancy [12]. Evidence from sub-group analyses of antenatal interventions also suggests that pregnant adolescents do not benefit as much as older pregnant women, potentially due to heightened health and nutrition needs that are not being met [13,14]. Overcoming these barriers is essential for ensuring healthy births and positive outcomes for pregnant adolescents. Developing tailored ANC for adolescents could address issues related to accessibility and support, improving attendance and increasing the likelihood of meeting their emotional, psychological, and physiological needs [15]. Successfully doing so could save lives and help break the intergenerational cycle of malnutrition.

Evidence remains limited on whether adolescents in low- and middle-income countries require tailored ANC services. Furthermore, it is unclear which specific services or adjustments would most effectively reduce the heightened risks of maternal and neonatal morbidity and mortality associated with adolescent pregnancies in these contexts. This report aimed to evaluate existing ANC services that are tailored to adolescents—whether to improve access to routine ANC or to provide enhanced or additional ANC services—and the impact they have on maternal and infant outcomes. The findings will contribute to informing what tailored ANC packages, if any, should be trialled to support optimal maternal and birth outcomes in pregnant adolescents in low- and middle-income countries (LMICs). To achieve this, the study had two related objectives: (1) to systematically review the literature on tailored antenatal care for adolescents; and (2) to gather insights from stakeholders working in LMICs on the relevance, availability, and components of tailored ANC for adolescents in their contexts.

## 2. Materials and Methods

### 2.1. Systematic Review

The systematic review was conducted following the Preferred Reporting Items for Systematic Reviews and Meta-Analyses (PRISMA) 2020 checklist (see Supplementary Material for checklist) [16]. The search strategy consisted of two key concepts: pregnant adolescents and ANC services. The full search strategy can be found in the Supplementary Material. Five databases were searched: MEDLINE, Embase, CINAHL (Cumulative Index to Nursing and Allied Health Literature), Global Health, and Cochrane Library. The initial search was performed in July 2023 and was then repeated in full in September 2024 by two reviewers.

#### 2.1.1. Inclusion and Exclusion Criteria

A ‘PICOSS’ framework (population, intervention, comparison, outcome, setting, study design) was used to determine the eligibility criteria of the studies reviewed (Table 1). Studies were excluded if they were non-human studies, not available in English, published prior to 2000, did not include adolescents (10–24 years), or were literature reviews, systematic reviews, or meeting reports. The age range was selected to be inclusive and follows the age range recommended by Sawyer et al. based on evidence that biological and social growth and development continue beyond the age of 19 years [15]. Due to limited evidence, all settings were included in order to draw lessons from all contexts. 

#### 2.1.2. Screening and Selection Process

Once the publications were collated, duplicates were removed. Titles and abstracts were then reviewed against the inclusion/exclusion criteria. For abstracts that appeared to meet the inclusion criteria, full texts were screened. Each study was assessed independently by two researchers for quality using the Methods for the Development of NICE quality appraisal tool [21]. Studies were rated ‘good’ (+++) if the study was designed or conducted in such a way as to minimise risk of bias (based on a list of criteria in the checklist—see reference for further details); ‘average’ (++) if aspects of the study are not clear or the study has addressed some but not all potential sources of bias; and ‘poor’ (+) if significant sources of bias where common.

#### 2.1.3. Data Extraction and Analysis

The data extracted included: title of study; first author; publication date; setting (location/region); year of study; study design; aim of study; recruitment of participants; age included; specific intervention or model of delivery; length of intervention; sample size; outcomes of interest; quality rating; and key findings and notes/comments. Due to the limited number of publications found, a meta-analysis was not possible. Therefore, a narrative synthesis was carried out to summarise and interpret the findings.

### 2.2. Survey

An anonymous, online, stakeholder survey was disseminated through the Global Adolescent Nutrition Network (GANN), which is a group of approximately 1000 individuals, mainly working in low- and middle-income countries (LMICs) (from 111 unique countries), and who have an interest in adolescent nutrition [22]. The survey consisted of three questions (which were designed by the authors but not tested for validity):

- Do you think that it is important for antenatal care for pregnant adolescents to include additional or altered support, compared to older pregnant adults, and/or be delivered in a youth-friendly manner?
- Expand on your answer by describing what specific elements (if any) you believe should be included in antenatal care for pregnant adolescents.
- In your context, do pregnant adolescents receive additional or tailored antenatal care? If yes, describe your context and the services being provided.

### 2.3. Data Analysis

Descriptive statistics were used to analyse the questions with binary yes/no options. Thematic analysis was conducted on the open-ended responses. Initial codes were generated from key concepts, which were then grouped into broader themes. These themes were refined and defined based on recurring patterns across responses, highlighting the specific elements of tailored ANC and examples of current service provision in different contexts. To provide further insight, the frequency of mentions for each theme was quantified, offering a clearer understanding of which aspects were most commonly emphasised by respondents.

## 3. Results

### 3.1. Systematic Review

**Figure 1:**
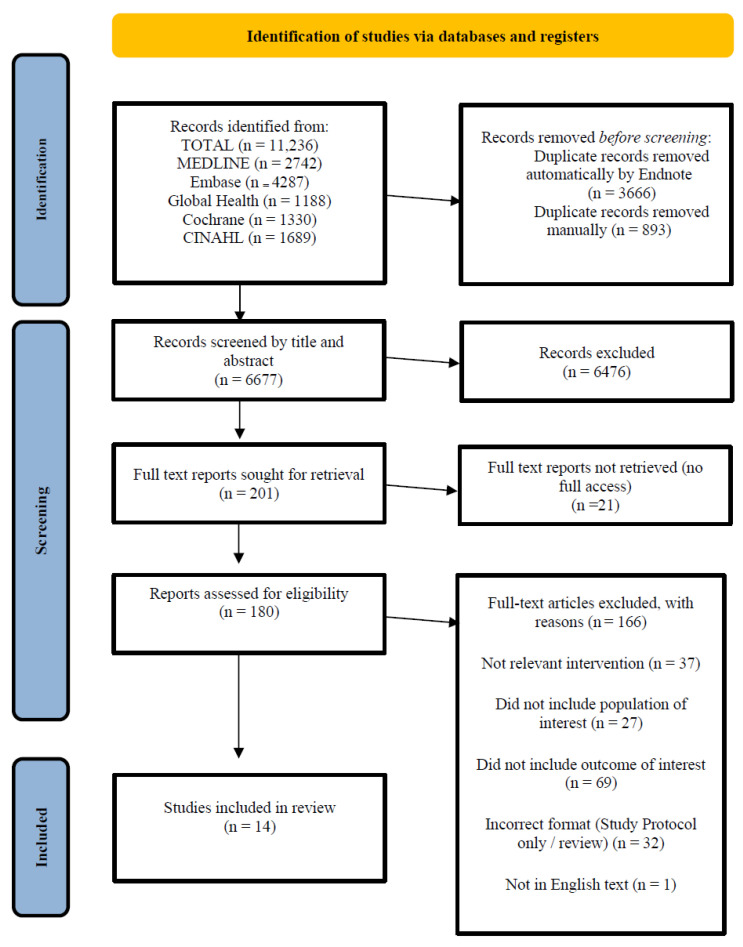
PRISMA flow diagram.

A total of 11,236 titles were identified through database searches. Once duplicates were removed, 6677 articles underwent the initial screening process (Figure 1). A total of 180 full-texts were reviewed and 14 publications were included in the results and assessed for quality [13,14,23,24]. All 14 studies reported infant birthweight outcomes; other outcomes reported included preterm birth, gestational age at delivery, and still-births (outcomes and characteristics for each study are described in Table 2).

The included studies were individually assessed for quality using the NICE quality appraisal tool [21]. For internal validity, six studies were given a good rating and eight studies were given an average rating. For external validity, two studies were given a good rating, eleven studies were given an average rating, and one was given a poor rating. Some studies were conducted among small populations with limited diversity, which impacted the grading (Table 3).

### 3.2. Study Interventions

The following sections provide a narrative description of the literature, presented by the type of ANC intervention for pregnant adolescents.

#### 3.2.1. Micronutrient Supplementation

Two studies examined the effects of micronutrient supplementation for adolescents during pregnancy [19,25]. Pinho-Pompeu et al. [19] conducted a cross-sectional study on the effect of WHO-recommended iron supplementation (40 mg twice daily) in anaemic pregnant adolescents on birth outcomes and found that untreated anaemic adolescents had significantly higher rates of preterm birth (*p* = 0.003), stillbirth (*p* = 0.004), and gestational age < 37 weeks (*p* = 0.036) compared to those who complied with treatment [19,34]. In a randomised controlled trial by Chan et al., 15- to 17-year-old pregnant adolescents who received a high dairy diet had greater intakes of calcium, phosphate, magnesium, and vitamin D than both controls (no supplementation) and the ‘orange juice fortified with calcium’ group. They also had significantly higher infant birth weights (*p* = 0.001) than both other groups [25].

#### 3.2.2. Supplementary Feeding

In a randomised controlled trial in Malawi, Friebert et al. assessed malnourished pregnant adults and adolescents (mid-upper arm circumference (MUAC) ≥20.6 and ≤23.0 cm) receiving one of two supplementary foods compared to standard care [13]. Adolescents who received one of the supplementary food interventions, either ready-to-use supplementary food (RUSF) or a fortified corn soy blend (CSB+) with a daily multiple micronutrient supplement, showed no significant differences in infant outcomes at birth, 6 weeks, or 12 weeks postpartum compared to those receiving standard care (*p* > 0.05) [13]. In addition, the subgroup analysis indicated that while supplementary foods improved some maternal health outcomes in adults, this was limited in adolescents [13]. Similarly, Koroma et al. conducted a secondary analysis from a randomised controlled trial in Sierra Leone to compare newborn outcomes with maternal age, following a supplementary feeding intervention in malnourished pregnant women (MUAC ≤ 23 cm) [14]. The trial compared the effect of maternal age on birth weight and found that adults receiving supplementary foods had infants with higher birth weights than adolescents who received the same supplementation (difference in difference: 166 g; 95% CI, 26–306; interaction *p* = 0.02) [14].

#### 3.2.3. Community-Based Delivery of ANC

The results of three studies showed that delivering ANC in the community was associated with improved infant and maternal outcomes [24,27,30]. Barnet et al. compared the outcomes between comprehensive adolescent pregnancy programmes (CAPPs) in school vs. hospital settings in the USA [24]. CAPPs must include standard ANC, primary, and preventative care for adolescent and infant, continuing care, nutrition services, educational services, mental health services, and referral to education and vocation services [24]. They found that adolescents in school-based CAPPs were significantly less likely to deliver LBW infants compared to those in hospital-based programmes (*p* = 0.006) [24]. Goyal et al. analysed data from the ‘Every Child Succeeds’ home visit programme in Ohio, USA, focusing on at-risk first-time mothers, with a mean maternal age of 20 years [27]. They found that a higher number of home visits was associated with reduced odds of preterm birth and small for gestational age (SGA); specifically, completing ≥ 8 visits by 26 weeks was linked to an adjusted odds ratio (aOR) of 0.38 for preterm birth and a hazard ratio (HR) of 0.32 for SGA [27]. Jacobs et al. conducted a randomised controlled trial providing tailored home visits to pregnant adolescents, also in the USA, which included goal-setting and health screenings, but found no significant impact on the ‘healthy birth’ indicator (birth weight above 2500 g and delivery at 37 weeks or more; OR 1.12, 95% CI: 0.71, 1.78) [30].

#### 3.2.4. Group-Delivery of ANC

Two studies reported on group-style ANC for pregnant adolescents [23,29]. A cluster randomised trial in New York (USA) health centres examined group prenatal care (8–12 pregnant adolescents per group), including reproductive health promotion versus individual care [29]. The trial, conducted in low-resource areas, showed that group care led to better birth, neonatal, and reproductive outcomes, with lower rates of LBW (8.7% vs. 9.8%) [29]. Although there was no difference in the total number of ANC visits between groups, attending more group prenatal care visits, as compared to individual visits, was associated with lower odds of delivering a small-for-gestational-age, preterm, or LBW infant [29]. A cohort study in Australia compared group ANC (referred to as caseload midwifery) to standard care at a clinic for young women/girls [23]. Using intention to treat analysis, group ANC significantly reduced preterm birth outcomes among adolescents compared to standard care (*p* < 0.014) but did not significantly impact LBW prevalence (*p* < 0.48) [23]. Those who received caseload midwifery were more likely to attend five or more antenatal visits compared to those in standard care (*p* = 0.002) [23].

#### 3.2.5. Tailored Nutrition Education

Two studies found nutrition education programmes to have a positive effect on outcomes [26,28]. A cohort study in USA reported on two tailored nutrition education programmes for pregnant adolescents, finding that the “Have a Healthy Baby” group had higher mean birth weights (3500 ± 600 g) and greater gestational weight gain (16.6 kg) compared to the “Eating Right is Basic” group (3200 ± 500 g; 14.8 kg) [28]. Both groups had low rates of preterm birth. The differences between these two curricula were not clear; however, the authors did state that ongoing support and follow-ups contributed to the positive outcome. In Bangladesh, a study comparing the effects of an antenatal ‘balanced plate’ nutrition education programme on pregnant adolescents versus adults found that adolescents experienced significantly higher birth weights (299.1 g; *p* = 0.003) compared to adults (95.6 g; *p* = 0.097) [26]. Additionally, the programme led to a greater reduction in LBW among adolescents (RR: 0.28; *p* = 0.007) compared to adults (RR: 0.54; *p* = 0.044) [26]. The programme was integrated into the existing community healthcare and included practical nutrition education sessions, and engagement sessions with family members.

#### 3.2.6. Additional Support and Counselling

Three studies examined the effects of tailored support and counselling on maternal and infant outcomes in adolescents [31,32,33]. Mersal et al. examined the effects of tailored antenatal counselling which focussed on improving pregnancy-related knowledge, health practices, and compliance with ANC at a maternal health centre in Egypt [32]. Adolescents that received standard care had poorer outcomes, with more preterm births (*p* = 0.001), LBW infants (*p* = 0.008), and stillbirths [32]. Jones et al. assessed the impact of a tailored adolescent antenatal service in the UK, which included a midwife-led clinic, home visits, and tailored education (individual or group) [31]. The named midwife for teenagers would then provide all ANC for those in a higher social risk category, which was determined using a referral proforma [31]. They reported an increase in birthweight for babies born to mothers participating in the programme, but due to the small sample size this was not statistically significant [31]. Root et al. demonstrated that participation in a tailored prenatal education and support programme, ‘Starting Out Right,’ reduced the odds of low birthweight by 30% (*p* ≤ 0.05) [33]. All three studies included age-specific support, which addressed the unique needs of pregnant adolescents.

Barnet et al. demonstrated the positive impact of psychosocial support in the form of health behaviour screening and advice on birth outcomes [24]. Comprehensive care, defined as ‘documentation of assessment and advice for psychosocial and behavioural conditions,’ was shown to have a much stronger association with low birth weight when compared to just adequate care (defined as care occurring by the fourth month of pregnancy) [24].

### 3.3. Stakeholder Survey

The survey received 102 responses, without the collection of any personal or identifiable data. Respondents were assumed to be professionals or experts familiar with adolescent health and nutrition, based on their membership of the Global Adolescent Nutrition Network (GANN).

All respondents agreed that pregnant adolescents require some form of tailored ANC. A majority of respondents (57%) indicated that providing both additional support and youth-friendly delivery of ANC are crucial for pregnant adolescents. A quarter of respondents (23%) said that pregnant adolescents require additional support over and above standard ANC, and 20% felt that youth-friendly delivery is important. However, the majority (78%) reported that, in their context, pregnant adolescents do not receive either form of tailored ANC.

Thematic analysis identified nine themes from the open-ended responses regarding which specific elements should be included in ANC for pregnant adolescents (Table 4), as well as what services currently exist in their contexts.

#### 3.3.1. Inclusive and Supportive Care

Thirty-one participants (30%) mentioned the need for inclusive and supportive care, highlighting the importance of judgement-free support and privacy.

*‘Adolescent girls need to be given extra care and attention because they usually face some social and emotional related issues like stigma.’*

*‘Giving them clear, youth-friendly information with encouragement that they need to make informed decisions and (feel as though) their provider is here for support.’*

*‘Allow them to have confidants accompany them into the appointments.’*

Many also suggested the need for specific training for health workers providing care for pregnant adolescents.

*‘It’s important to not just think about how support can be altered for the adolescent mother, but also how we’re training healthcare workers to provide non-judgmental, respectful, supportive care to adolescent mothers. Adolescent mothers face a lot of stigma and abuse in the community, including from health workers.’*

#### 3.3.2. Tailored Health Education

Twenty-eight respondents (27%) highlighted the importance of providing tailored health education as part of ANC. Sexual and reproductive health (SRH) education was often considered a key element.

*‘Have targeted health talk sessions for pregnant adolescent girls rather than a blanket one in areas such as importance of iron and folic acid supplementation, danger signs, childbirth, child stimulation and parenting.’*

*‘Adolescents may have limited knowledge of reproductive health, pregnancy, and childbirth. ANC should incorporate comprehensive sexual and reproductive health education to inform them about safe pregnancy practices, birth planning, postpartum care, and future contraception. Information and advice should be delivered in a way that resonates with adolescents, using engaging, age-appropriate communication tools, and offering visual and interactive learning experiences.’*

#### 3.3.3. Tailored Nutrition Care

Twenty-three respondents (23%) mentioned the importance of tailoring nutritional care to the specific needs of pregnant adolescents. This included considering the macro- and micro-nutrient needs of adolescents as well as screening for deficiencies.

*‘ANC should not only address the current pregnancy nutrition needs but also ensure that they achieve their required growth and meet their specific nutrition requirements. By providing such tailored nutrition support, we can help these young mothers reach their full potential despite the dual demands of increased growth spurt and pregnancy on their physiology.’*

*‘Given the higher risks associated with adolescent pregnancy, I think that pregnant adolescent girls might require more follow up and/or additional tests & subsequent support. For example, ensuring that adolescent girls are not micronutrient deficient/anaemic and providing proper support through supplementation.’*

#### 3.3.4. Mental Health Support and Counselling

Twenty-two respondents highlighted the need for mental health support and counselling during pregnancy, often mentioning the rise in mental health conditions among adolescents. Additionally, they stressed the importance of screening for mental health conditions as part of ANC.

*‘Psychosocial counselling for adolescent girls is to be added as this is an unexpected responsibility for them at quite a young age.’*

*‘Addressing mental health is crucial, as adolescent pregnancies can lead to feelings of isolation and stress.’*

Other key elements identified included support beyond birth, partner and family engagement, youth only times at clinics, peer support, and flexible service locations.

#### 3.3.5. Existing Tailored Antenatal Care

There were limited responses regarding what is currently being included in respondents’ contexts. The most frequently mentioned services were:

- Tailored nutrition care, particularly supplementation (nine mentions)
- Tailored educational support, which included nutrition and SRH education (seven mentions)
- Mental health support and counselling (seven mentions)
- Support beyond birth, such as assistance in returning to education and livelihood programmes (five mentions).

**Figure 2:**
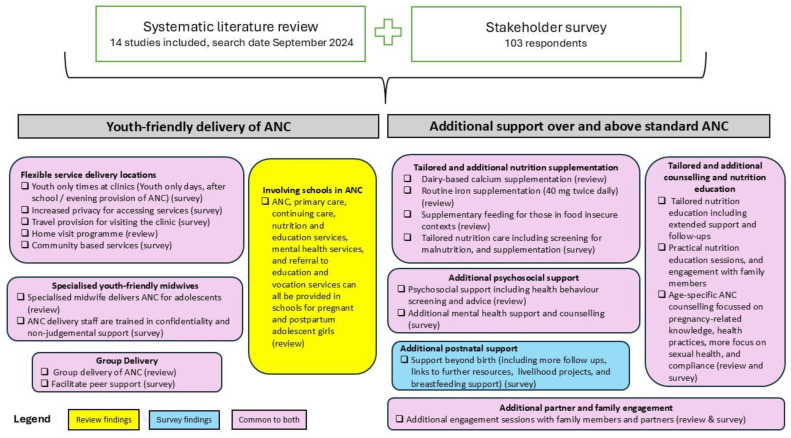
Overview of results: literature review and stakeholder survey.

Other examples mentioned (four times or less) included peer support, youth-only clinic times, inclusive and supportive care, partner and family engagement, and flexible service locations. An overview of the results from both the survey and the literature review can be found in Figure 2.

## 4. Discussion

The findings from both the systematic review and the survey highlight the importance of developing adolescent-friendly ANC services for pregnant adolescent girls to improve maternal and infant health outcomes. Evidence from the review indicates that youth-friendly delivery is important, and that tailoring some aspects of antenatal support, especially counselling and nutrition education, can improve maternal and infant birth outcomes [28,31,32,33]. There is also some evidence to suggest that additional micronutrient supplements, not just iron supplements, may benefit this age group [25], and that macronutrient supplementation dosages may need to be higher than that of adult women in order to see a benefit [14].

Successful youth-friendly delivery mechanisms seen in the literature include group-based and community-focused ANC models for pregnant adolescents [20,23,24,27,29]. These approaches can provide a support system for pregnant adolescents alongside the delivery of other services and educational messages, in a comfortable, accepting setting [35]. In addition, community-based care may be a way of addressing some of the accessibility barriers often overlooked for pregnant adolescents [24] and can improve the sustainability of programmes [26].

The evidence from the review suggests that providing routine micronutrient supplementation in line with WHO recommendations can improve maternal and infant birth outcomes for adolescents [19]. Improving access for adolescent girls to routine ANC services would reap these benefits. Beyond iron supplementation, one study showed that other micronutrients are also beneficial [25], suggesting the potential need to prioritise multiple micronutrient supplements (MMS) in this age group. The benefits of MMS on birth outcomes are also well evidenced for adult mothers; however, this is yet to be recommended by WHO as routine [36]. There is an urgent need for more research into the benefits of MMS and heightened macronutrient supplementation in this age group [1,37].

Other additional ANC support that was evidenced in the literature was additional counselling on health during pregnancy, SRH, and compliance to interventions; as well as more practical nutrition education sessions, more psychosocial support and mental health interventions, and ANC sessions that also engage family members and partners. Similar suggestions were raised by respondents of the stakeholder survey, especially around additional mental health support. Adolescent mothers face significantly higher rates of depression, both prenatally and postpartum, compared to adult mothers and their nonpregnant peers [38]. Implementing routine screening for mental health conditions, as suggested by survey participants, could facilitate early intervention and support, potentially mitigating the long-term consequences of poor mental health on both mother and child. However, the survey also suggested that coverage of tailored ANC for pregnant adolescent girls is currently very low, highlighting a gap between the identified needs and the existing services for this group.

Given the limited resources for health services in many settings, especially LMICs, there will be a need to consider whether additional ANC services are cost-effective, over and above ensuring that pregnant adolescent girls access routine ANC. Staff training costs and resource constraints would need to be considered. While evidence is currently lacking for much of this area, the importance of providing inclusive, supportive, judgement-free, and confidential care was clear from both the survey and literature [12,39,40]. Stigma and shame often prevent pregnant adolescents from seeking the care they need and from accessing care early on in pregnancy [41].

### 4.1. Strengths

The addition of survey findings to this systematic literature review provides unique insights into stakeholder views, complementing and contextualising the other results, especially since the objectives of our review were to inform the testing of future ANC packages in LMICs, whereas much of the literature found was only from high-income settings. The survey highlights strong support for tailored ANC and identifies critical components, reinforcing the evidence from the included studies. The review, while only finding a limited number of papers, does highlight examples of studies that have evaluated ANC interventions specifically for pregnant adolescents as a separate, more vulnerable group, setting a precedent for future work.

### 4.2. Limitations

While the individual interventions reviewed showed largely positive effects on maternal and infant birth outcomes, the limited number of studies and lack of standardisation among interventions limited our ability to pool and compare studies. We did not assess the certainty of the evidence using ‘GRADE’ since more research is needed for policy recommendations to be made. There is a particular need for future studies to differentiate between younger and older adolescents (e.g., those <16 years vs. ≥16 year) since the risks are likely to be very different and pooling is likely to dilute or obscure these. There is also need for more high-quality, large sample size, randomised study designs. The review was also only limited to antenatal interventions and infant birth outcomes, whereas future systematic reviews should expand on these results to include postnatal services for adolescents and other maternal outcomes beyond birth. In addition, since most studies included in this review were conducted in high-income countries, it is important to note that some may not be relevant or generalisable to LMICs. A methodological limitation of our review was the lack of prior public registration of the search protocol, and the lack of inclusion of non-English-language papers. For the survey, there is a risk of non-response bias since only approximately 10% of those invited to take the survey submitted responses. In addition, due to limited access, pregnant adolescents were not surveyed, excluding a key stakeholder group and potentially limiting the study’s comprehensiveness.

## 5. Recommendations for Future Research

Intervention trials are needed to test a future standard ANC package for pregnant adolescents that includes the key elements outlined in this report. Such a package should be contextualised to the setting following a review of the current services provided, and should be co-designed with input from adolescent girls themselves [42]. The study should be designed to assess the cost-benefits of additional services vs. simply improving access to routine services. An effective package of tailored ANC services for pregnant adolescents could significantly contribute to reducing maternal deaths, improving new born survival, reducing childhood undernutrition, supporting adolescent mothers to reach their full physical and mental potential, and breaking the intergenerational cycle of malnutrition.

## 6. Conclusions

The results of the systematic review, backed by survey data, are an important starting point for identifying key elements of adolescent-tailored ANC. These include support and counselling (including mental health), health education (including SRH), greater engagement of family members, and nutrition care (macro- and micro-nutrient supplementation, dietary advice, and practical nutrition education). The services should be delivered in a youth-friendly manner, including non-judgemental care, increased privacy, group sessions, and practical considerations for access such as location, timing and cost. Trialling such packages against standard ANC should be a priority in order to gain adequate evidence to update national and global ANC guidelines, ultimately better reflecting the needs of pregnant adolescent girls.

## Figures and Tables

**Table 1:** PICOSS framework of eligibility criteria.

Population	Adolescent girls up to age 24 years of age (while the WHO defines adolescence as 10–19 years, we opted for the broader Sawyer et al, definition since the literature was limited)
Intervention	Tailored antenatal care services for pregnant adolescents
Comparison	No comparator required
Outcome	Maternal and infant birth outcomes: Birth weight: body weight of infant at birth [17] Premature Birth: infants born alive before 37 weeks of pregnancy [18] Gestational age at birth: measure of the pregnancy taken from the beginning of the women’s last menstrual cycle [19] Still Birth: baby who dies before or during birth, but after 28 weeks of pregnancy [20]
Setting	Any setting will be included
Study Design	Any study design will be considered

**Table 2:** Characteristics and outcomes of included studies (*n* = 14).

Author(s)	Title of Study	Study Design	Setting	Population Characteristics	Exposure	Outcome(s) Reported	Findings
Allen et al. (2015) [23]	Does model of maternity care make a difference to birth outcomes for young women? A retrospective cohort study	Cohort Study (mixed method evaluation)	Australia	Age < 21 years, *n* = 1908	Three groups: Group prenatal care and home visits ANC in a community ‘young women’s clinic’ Standard care (control) visits in health clinic or hospital	Birth weight Preterm birth Gestational age Still births	Preterm decreased in group care vs. standard (ITT) (*p* = 0.014) LBW decreased (*p* = 0.049 (per protocol only) in Young Women’s Clinic vs. standard care (positive)
Barnet et al. (2003) [24]	Reduced Low Birth Weight for Teenagers Receiving Prenatal Care at a School-based Health Center: Effect of Access and Comprehensive Care	Retrospective Cohort Study	Baltimore, Maryland, USA	Age 11–18 years, *n* = 390	Comprehensive adolescent pregnancy programmes (CAPP) in two different settings - School-based (at alternative public school for pregnant teens) - Hospital-based	Birth weight	Those receiving the school-based CAPP were less likely to deliver a LBW infant than those receiving hospital-based CAPP (*p* = 0.006) (positive)
Chan et al. (2006) [25]	Effects of Dietary Calcium Intervention on Adolescent Mothers and Newborns	Randomised Controlled Trial	Utah, USA	Age 15–17 years, Enrolled before 20 weeks of gestation, *n* = 72	Calcium supplementation Dairy Products (milk, yogurt, cheese equalling > 1200 mg Ca) Orange juice fortified with calcium (>1200 mg total Ca) Control (no additional Ca)	Birth weight Gestational age at birth	Infants (3517 ± 273 g) in the dairy group were heavier than infants in the control (3277 ± 177 g) and orange juice plus calcium (3292 ± 165 g) groups. *p* < 0.00. (positive) Gestational age at birth wasn’t affected.
Chowdhury, M., et al. (2022) [26]	The impact of antenatal balanced plate nutrition education for pregnant women on birth weight: a cluster randomised controlled trial in rural Bangladesh.	Cluster Randomised Controlled Trial	Bangladesh	Age 15–49 years (separate post-hoc analysis for 15–20-year olds); gestation 12 weeks or less *n* = 893	Intervention group: antenatal education based on a balanced plate nutrition intervention vs. standard ANC	Birthweight	Birthweight was greater in adolescent mothers compared to adults (299 g vs. 96 g) Reduction in %LBW was greater in adolescents compared to adults. (RR: 0.28; 95% CI: 0.11, 0.71; vs. RR: 0.54; 95% CI: 0.29, 0.98;) (positive)
Friebert, A., et al. (2017) [13]	Adolescent pregnancy and nutrition: a subgroup analysis from the ‘Mama chiponde’ study in Malawi	Randomised Controlled Trial	Malawi	18+ years with subgroup analysis for age 18–20 years; fundal height < 35 cm, *n* = 1828	Ready-to-use supplementary food (RUSF) or a fortified corn soy blend (CSB+) with a daily multiple micronutrient antenatal supplement compared to standard care	Birthweight Gestational age	There were no differences in infant outcomes at birth or at 6- and 12- week postpartum across treatment groups (*p* > 0.05). (null)
Goyal, N. K., et al. (2013) [27]	Dosage effect of prenatal home visiting on pregnancy outcomes in at-risk, first-time mothers.	Retrospective Cohort Study	Ohio, USA	First time mothers (mean maternal age 20 years), gestation 26 weeks or less, *n* = 441	Varying doses of intervention (including none) Prenatal home visits using Every Child Succeeds (ECS) model. Visits provided by social workers, child development specialists, and nurses. Weekly or more frequent visits and tapering to fewer visits as the child ages	Birthweight Preterm birth Gestational age	Completion of ≥8 home visits by 26 weeks was associated with reduced odds of preterm birth (95% CI: 0.16–0.87). Completion of ≥12 home visits was associated reduced hazard ratio of SGA (95% CI: 0.15–0.68) (positive)
Hunt et al. (2002) [28]	Effects of nutrition education programmes on anthropometric measurements and pregnancy outcomes of adolescents	Cohort Study	Oklahoma, USA	Age 14–19 years, *n* = 32	“Eating Right is Basic” and “Have A Healthy Baby” interventions (both 8-week nutrition education programmes)	Birth weight Preterm birth	The “Have a Healthy Baby” group had higher mean birth weights (3500 ± 600 g) and greater weight gain (16.6 kg) compared to the “Eating Right is Basic” group (3200 ± 500 g; 14.8 kg). (positive)
Ickovics et al. (2016) [29]	Cluster Randomized Controlled Trial of Group Prenatal Care: Perinatal Outcomes Among Adolescents in New York City Health Centers	Cluster Randomised controlled trial	New York City, USA	Age 14–21 years, gestational age less than 24 weeks, *n* = 1233	Group ANC vs. individual standard care	Birth weight Preterm birth	Pregnant adolescents who attended group ANC were significantly less likely to have infants small for gestational age (<10th percentile; 11.0% vs. 15.8%; ORL 0.66; 95% CI: 0.44, 0.99). (positive) More group care visits lowered the odds of SGA (OR 0.91, 95% CI 0.85,0.99), LBW (OR 0.81, 95% CI 0.73, 0.89), and preterm (OR 0.76, 95% CI 0.69, 0.84)
Jacobs, F., et al. (2016) [30]	Improving adolescent parenting: results from a randomised controlled trial of a home visiting programme for young families.	Randomised Controlled Trial	Massachusetts, USA	Age 16–21 years, *n* = 704	Intervention group: Healthy Families Massachusetts home visiting services; includes curriculum-based activities and linkages to other services Vs standard ANC	Birthweight	No significant effect was seen as a result of taking part in the programme. (null)
Jones., et al. (2016) [31]	Is the introduction of a named midwife for teenagers associated with improved outcomes? A service development project.	Retrospective Cohort Study	Surrey, UK	Age ≤ 19 years, *n* = 83	Intervention group received midwife-led teenage ANC, home visits, and tailored education Vs standard ANC	Birth weight Gestational age	Infants born to the intervention group had no LBW cases (<2500 g) and a higher proportion of infants in the healthy weight range (2500–3999 g) compared to adolescents that didn’t receive the intervention. (positive) There was no significant difference in average gestational age
Koroma et al. (2023) [14]	Supplementary feeding and infection control in pregnant adolescents— A secondary analysis of a randomised trial among malnourished women in Sierra Leone	Randomised controlled trial	Sierra Leone	All pregnant women, with subgroup analysis of age groups <18, 18–19, >20; median time from enrolment to delivery = 16 weeks; *n* = 1431, <18 (*n* = 236), 18–19 (*n* = 454)	Intervention group received azithromycin, testing for vaginal dysbiosis, daily ration of ready-to-use supplemental food (520 kcal, 18 g protein, over 100% of recommended micronutrients). Control group received standard care, 250 g/day of corn soy blended flour, 25 g of palm oil daily (589 kcal, 17.5 g protein), iron, folic acid	Birth weight Still birth	Adults benefited more than younger adolescents with respect to birth weight (difference in difference, 166 g; 95% CI, 26, 306), birth length (difference in difference, 7.4 mm; 95% CI, 0.1, 14.8), and risk for LBW (<2.5 kg) (interaction *p* = 0.019). (negative)
Mersal et al. (2013) [32]	Effect of prenatal counselling on compliance and outcomes of teenage pregnancy	Randomised Control Study (mixed methods)	Egypt	Age 15–18 years, enrolled in second trimester, *n* = 86	Tailored prenatal counselling Vs standard care	Birth weight Preterm birth Still births	Intervention group had significantly higher birth weight (*p* = 0.008) and lower risk of preterm birth (*p* < 0.001) (positive)
Pinho-Pompeu et al. (2017) [19]	Anemia in pregnant adolescents: impact of treatment on perinatal outcomes	Cross-Sectional Study	Brazil	Age 10–19 years, *n* = 458	Oral iron supplementation (elemental iron 40 g twice/day) vs. no supplementation	Birth weight Preterm birth Gestational age Still births	No supplementation with iron (untreated) was associated with preterm birth (*p* = 0.003), gestational age < 37 weeks (*p* = 0.036) and still births (*p* = 0.004). (positive)
Root, A.D., et al. (2023) [33]	Health Characteristics and Birth Outcomes for “Starting Out Right,” a Teen Pregnancy Program	Observational Cohort Study	Arizona, USA	Age ≤19 years, *n* = 12,586	Intervention group received prenatal education and support programme for pregnant teens, including 8 structured in-person classes, with case management and support groups, vs. standard ANC	Birth weight Preterm birth	Intervention associated with 30% lower odds of LBW (95% CI 0.6–0.9). (positive) Intervention associated with 10% lower odds of preterm birth (95% CI 0.7–1.1).

LBW = low birth weight; SGA: small for gestational age; ANC = antenatal care.

**Table 3:** Quality rating of studies.

Author(s)	Quality Rating	
	Internal Validity	External Validity
Allen et al. (2015) [23]	+	+
Barnet et al. (2003) [24]	+	+
Chan et al. (2006) [25]	+	+
Chowdhury et al. (2022) [26]	++	++
Friebert, et al. (2017) [13]	+	+
Goyal, et al. (2013) [27]	++	+
Hunt et al. (2002) [28]	+	+
Ickovics et al. (2016) [29]	++	+
Jacobs et al. (2016) [30]	++	+
Jones., et al. (2016) [31]	+	-
Koroma et al. (2023) [14]	+	+
Mersal et al. (2013) [32]	++	+
Pinho-Pompeu et al. (2017) [19]	+	+
Root et al. (2023) [33]	++	++

+ = poor quality rating, ++ = average quality rating (see further explanation in Section 2).

**Table 4:** Results from the stakeholder survey; specific elements to be included in tailored antenatal care for adolescents.

Type of Tailored ANC	Theme	Codes	Mentions (n)
Youth-friendly delivery	Inclusive and supportive care	Privacy, training for healthcare workers, non-judgemental support	31
Additional services	Tailored educational support	Age specific health and nutrition education, SRH education	28
Additional services	Tailored nutrition care	Supplementation, screening	23
Additional services	Mental health support and counselling	Mental health counselling, psychosocial support	22
Additional services	Support beyond birth	Follow ups, links to further resources, livelihood projects, postnatal care, breastfeeding support	16
Youth-friendly delivery	Youth only times at clinics	Youth only days, after school/evening provision of ANC	9
Additional services	Partner and family engagement	Family engagement, partner engagement	9
Additional services	Peer support	Support groups, peer educators	6
Youth-friendly delivery	Flexible service locations	Community based services, adolescent only clinics, travel provision	5

## Data Availability

No new data were created or analysed in this study.

## Supplementary Materials

The following supporting information can be downloaded at: https://www.mdpi.com/article/10.3390/nu18050727/s1, Table S1: Full search strategy; Table S2: PRISMA checklist.
