# Supplementary material for: Informing the Development of Tailored Antenatal Care Services for Pregnant Adolescents: A Systematic Review and Stakeholder Survey
Source: Nutrients. 2026 Feb 24;18(5):727. doi: 10.3390/nu18050727 (PMC12987006; doi:10.3390/nu18050727)
Supplement: Supplementary file 1 [file nutrients-18-00727-s001.zip › nutrients-4115092-supplementary.pdf]

Table S1. Full search strategy

MEDLINE			
**Search #**	**Terms**	**Hits – 1st Review**	**Hits - 2nd Review**
1	exp Maternal Health Services/	57842	60181
2	maternal health services/ or reproductive health services/	18657	19546
3	((maternal or pregnancy or childbirth) adj3 (service* or health or education or program* or care or intervention* or counsel*)).mp.	70847	77988
4	((adolescent* or teen or teens or teenager* or young person* or young people* or youth or youths or "10-19 year* old" or "10 to 19 year* old" or "age* 10 to 19" or "age* 10-19" or girl or girls) adj3 (pregnan* or childbearing or mother*)).mp.	12589	13297
5	(perinatal care or perinatal or postnatal care or postnatal or prenatal or prenatal care or ANC or PNC or antenatal care or antenatal).mp.	387216	407755
6	1 or 2 or 3 or 5	435591	460695
7	Adolescent Pregnancy/	8477	8676
8	4 or 7	16338	17096
9	6 and 8	4045	4253
10	limit 9 to (english language and humans and yr="2000 - Current")	2245	2742

Embase			
**Search #**	**Terms**	**Hits – 1st Review**	**Hits – 2nd review**
1	exp Maternal Health Services/	2868	3355
2	Reproductive health services/	181299	187338
3	((maternal or pregnancy or childbirth) adj3 (service* or health or education or program* or care or intervention* or counsel*)).mp.	90956	99507

4	((adolescent* or teen or teens or teenager* or young person* or young people* or youth or youths or "10-19 year* old" or "10 to 19 year* old" or "age* 10 to 19" or "age* 10-19" or girl or girls) adj3 (pregnan* or childbearing or mother*)).mp.	20122	20938
5	(perinatal care or perinatal or postnatal care or postnatal or prenatal or prenatal care or ANC or PNC or antenatal care or antenatal).mp.	586829	612871
6	1 or 2 or 3 or 5	812659	851221
7	Adolescent Pregnancy/	10162	10835
8	4 or 7	20122	20938
9	6 and 8	5472	5749
10	limit 9 to (english language and humans and yr="2000 - Current")	3859	4287

**Global Health**			
**Search #**	**Terms**	**Hits – 1st review**	**Hits – 2nd review**
1	Maternal Health Services	893	1017
2	Reproductive health services	52	1417
3	((maternal or pregnancy or antenatal or postpartum or prenatal) adj3 (service* or health or education or program* or care or intervention*))	36129	40431
4	((adolescent* or teen or teens or teenager* or young person* or young people* or youth or youths or "10-19 year* old" or "10 to 19 year* old" or "age* 10 to 19" or "age* 10-19" or girl or girls) adj3 (pregnan* or childbearing or mother*))	4361	4721
5	Adolescent Mothers	503	545
6	(perinatal care or perinatal or postnatal care or postnatal or prenatal or prenatal care or ANC or PNC or antenatal care or antenatal)	69193	74475
7	1 or 2 or 3 or 6	85639	93882

8	4 or 5	4361	4721
9	7 and 8	1233	1417
10	limit 9 to yr="2000 -Current"	1164	1349
11	limit 10 to english language	1010	1188

**Cochrane Library**			
**Search #**	**Terms**	**Hits – 1st review**	**Hits – 2nd review**
1	[mh "maternal health services"]	3219	3544
2	[mh ^"pregnancy in adolescence"]	257	295
3	(("maternal" or "pregnancy" or "childbirth") NEXT/3 (service* or health or education or program* or care or intervention*))	8379	9402
4	[mh ^"reproductive health services"]	52	73
5	((adolescent* or teen or teens or teenager* or young NEXT person* or young NEXT people* or youth or youths or age* or girl or girls or "10 to 19 year old" or "age 10 to 19 year old") NEXT/3 (pregnan* or childbearing or mother*))	4549	5105
6	(perinatal care or perinatal or postnatal care or postnatal or prenatal or prenatal care or ANC or PNC or antenatal care or antenatal)	26095	28755
7	#1 OR #3 OR #4 OR #6	30578	33754
8	#2 OR #5	4652	5221
9	#7 AND #8 In	1171	1361
10	#9 English only	1143	1330

**CINAHL**			
**Search #**	**Terms**	**Hits – 1st review**	**Hits – 2nd review**
1	(MH "Preventive Health Care") OR (MH "Prepregnancy Care") OR (MH "Prenatal Care") OR (MH "Postnatal Care")	50852	53567

2	((maternal or pregnancy or childbirth) N3 (service* or health or education or program* or care or intervention* or counsel*))	50822	53554
3	((adolescent* or teen or teens or teenager* or young person* or young people* or youth or youths or "10-19 year* old" or "10 to 19 year* old" or "age* 10 to 19" or "age* 10-19" or girl or girls) N3 (pregnan* or childbearing or mother*))	8545	8846
4	perinatal care or perinatal or postnatal care or postnatal or prenatal or prenatal care or ANC or PNC or antenatal care or antenatal	136926	137803
5	(MH "Pregnancy in Adolescence") OR (MH "Maternal Age 14 and Under")	5908	6045
6	S1 OR S2 OR S4	193717	197708
7	S3 OR S5	11431	11805
8	S6 AND S7	3048	3137
9	Limit 8 - 2000 to current, Humans only, English only	1600	1689

Table S2. PRISMA Checklist (*From:* Page MJ, McKenzie JE, Bossuyt PM, Boutron I, Hoffmann TC, Mulrow CD, et al. The PRISMA 2020 statement: an updated guideline for reporting systematic reviews. BMJ 2021;372:n71. doi: 10.1136/bmj.n71. This work is licensed under CC BY 4.0. To view a copy of this license, visit https://creativecommons.org/licenses/by/4.0/)

Section and Topic	Item #	Checklist item	Location where item is reported
**TITLE**			
Title	1	Identify the report as a systematic review.	Title and methods section
**ABSTRACT**			
Abstract	2	See the PRISMA 2020 for Abstracts checklist.	
**INTRODUCTION**			
Rationale	3	Describe the rationale for the review in the context of existing knowledge.	Introduction section
Objectives	4	Provide an explicit statement of the objective(s) or question(s) the review addresses.	Last paragraph of the introduction section
**METHODS**			
Eligibility criteria	5	Specify the inclusion and exclusion criteria for the review and how studies were grouped for the syntheses.	Subheading within methods section
Information sources	6	Specify all databases, registers, websites, organisations, reference lists and other sources searched or consulted to identify studies. Specify the date when each source was last searched or consulted.	1st paragraph of methods section
Search strategy	7	Present the full search strategies for all databases, registers and websites, including any filters and limits used.	Supplementary material
Selection process	8	Specify the methods used to decide whether a study met the inclusion criteria of the review, including how many reviewers screened each record and each report retrieved, whether they worked independently, and if applicable, details of automation tools used in the process.	Methods section
Data collection process	9	Specify the methods used to collect data from reports, including how many reviewers collected data from each report, whether they worked independently, any processes for obtaining or confirming data from study investigators, and if applicable, details of automation tools used in the process.	'Data extraction' subheading within methods section, and throughout methods

Section and Topic	Item #	Checklist item	Location where item is reported
			section
Data items	10a	List and define all outcomes for which data were sought. Specify whether all results that were compatible with each outcome domain in each study were sought (e.g. for all measures, time points, analyses), and if not, the methods used to decide which results to collect.	Outcomes listed in Table 1
	10b	List and define all other variables for which data were sought (e.g. participant and intervention characteristics, funding sources). Describe any assumptions made about any missing or unclear information.	listed under 'data extraction' section
Study risk of bias assessment	11	Specify the methods used to assess risk of bias in the included studies, including details of the tool(s) used, how many reviewers assessed each study and whether they worked independently, and if applicable, details of automation tools used in the process.	'Screening and selection process' subheading within methods
Effect measures	12	Specify for each outcome the effect measure(s) (e.g. risk ratio, mean difference) used in the synthesis or presentation of results.	Data extraction and analysis section explains that a narrative synthesis was used
Synthesis methods	13a	Describe the processes used to decide which studies were eligible for each synthesis (e.g. tabulating the study intervention characteristics and comparing against the planned groups for each synthesis (item #5)).	All included studies were synthesised
	13b	Describe any methods required to prepare the data for presentation or synthesis, such as handling of missing summary statistics, or data conversions.	Not applicable
	13c	Describe any methods used to tabulate or visually display results of individual studies and syntheses.	Not applicable
	13d	Describe any methods used to synthesize results and provide a rationale for the choice(s). If meta-analysis was performed, describe the model(s), method(s) to identify the presence and extent of statistical heterogeneity, and software package(s) used.	Not applicable
	13e	Describe any methods used to explore possible causes of heterogeneity among study results (e.g. subgroup analysis, meta-regression).	Not applicable
	13f	Describe any sensitivity analyses conducted to assess robustness of the synthesized results.	Not applicable
Reporting bias assessment	14	Describe any methods used to assess risk of bias due to missing results in a synthesis (arising from reporting biases).	Not applicable
Certainty assessment	15	Describe any methods used to assess certainty (or confidence) in the body of evidence for an outcome.	Not applicable

Section and Topic	Item #	Checklist item	Location where item is reported
**RESULTS**			
Study selection	16a	Describe the results of the search and selection process, from the number of records identified in the search to the number of studies included in the review, ideally using a flow diagram.	First paragraph of results, and Figure 1 (flow diagram)
	16b	Cite studies that might appear to meet the inclusion criteria, but which were excluded, and explain why they were excluded.	Figure 1 (flow diagram)
Study characteristics	17	Cite each included study and present its characteristics.	Table 2
Risk of bias in studies	18	Present assessments of risk of bias for each included study.	Table 3
Results of individual studies	19	For all outcomes, present, for each study: (a) summary statistics for each group (where appropriate) and (b) an effect estimate and its precision (e.g. confidence/credible interval), ideally using structured tables or plots.	Outcomes presented in narrative format in results section and in Table 2
Results of syntheses	20a	For each synthesis, briefly summarise the characteristics and risk of bias among contributing studies.	Not applicable
	20b	Present results of all statistical syntheses conducted. If meta-analysis was done, present for each the summary estimate and its precision (e.g. confidence/credible interval) and measures of statistical heterogeneity. If comparing groups, describe the direction of the effect.	Not applicable
	20c	Present results of all investigations of possible causes of heterogeneity among study results.	Not applicable
	20d	Present results of all sensitivity analyses conducted to assess the robustness of the synthesized results.	Not applicable
Reporting biases	21	Present assessments of risk of bias due to missing results (arising from reporting biases) for each synthesis assessed.	Not applicable
Certainty of evidence	22	Present assessments of certainty (or confidence) in the body of evidence for each outcome assessed.	Not applicable
**DISCUSSION**			
Discussion	23a	Provide a general interpretation of the results in the context of other evidence.	Discussion section
	23b	Discuss any limitations of the evidence included in the review.	Discussion section
	23c	Discuss any limitations of the review processes used.	Discussion section

Section and Topic	Item #	Checklist item	Location where item is reported
	23d	Discuss implications of the results for practice, policy, and future research.	Discussion section
**OTHER INFORMATION**			
Registration and protocol	24a	Provide registration information for the review, including register name and registration number, or state that the review was not registered.	The review was not registered
	24b	Indicate where the review protocol can be accessed, or state that a protocol was not prepared.	A protocol was not prepared
	24c	Describe and explain any amendments to information provided at registration or in the protocol.	Not applicable
Support	25	Describe sources of financial or non-financial support for the review, and the role of the funders or sponsors in the review.	Page 23 – Funding statement
Competing interests	26	Declare any competing interests of review authors.	Page 23 – Conflict of Interest
Availability of data, code and other materials	27	Report which of the following are publicly available and where they can be found: template data collection forms; data extracted from included studies; data used for all analyses; analytic code; any other materials used in the review.	Data availability statement
